# Amyopathic Dermatomyositis in a Patient With Breast Cancer in Remission: A Case Report and a Systematic Review of the Literature

**DOI:** 10.1155/crdm/9193986

**Published:** 2025-09-28

**Authors:** Rym Afiouni, Reine Merhy, Maria Farhat, Hampig Kourie, Roland Tomb

**Affiliations:** ^1^Department of Dermatology, Hôtel-Dieu de France University Hospital, Saint Joseph University, Beirut, Lebanon; ^2^Department of Oncology, Hôtel-Dieu de France University Hospital, Saint Joseph University, Beirut, Lebanon

**Keywords:** amyopathic dermatomyositis, breast cancer, dermatomyositis, remission, skin

## Abstract

Amyopathic dermatomyositis is a rare inflammatory disease in the spectrum of dermatomyositis characterized by typical skin lesions in the absence of muscle involvement. Dermatomyositis is a well-known paraneoplastic syndrome that can reveal or indicate a relapse of an underlying malignancy. To our knowledge, only a few cases of amyopathic dermatomyositis associated with breast cancer have been described in the literature. Here, we describe the case of a 52-year-old female with known triple-negative breast cancer in remission presenting with amyopathic dermatomyositis with a good response to hydroxychloroquine, and we performed a systematic review of this disease association. These patients should be followed closely for skin manifestations recurrence, therefore cancer recurrence or a new malignancy diagnosis.

## 1. Introduction

Dermatomyositis (DM) is a rare inflammatory myopathy characterized by classical cutaneous manifestations and proximal myopathy. Amyopathic DM is a less frequent disease in the spectrum of DM in which there is no muscle involvement [1]. Only a few cases of amyopathic DM associated with breast cancer have been in the literature [1]. We present the case of a female patient with a known triple negative breast cancer in remission diagnosed with amyopathic DM.

## 2. Case Presentation

A 52-year-old woman presented to our dermatology department for pruritic cutaneous eruption on the face and hands. There was no family history of autoimmune disease. The patient had a medical history of a triple-negative locally advanced breast cancer diagnosed in April 2018. She was treated with dose-dense neoadjuvant chemotherapy, 4 cycles of doxorubicin/cyclophosphamide and 12 injections of paclitaxel weekly, followed by a modified radical mastectomy with lymph node dissection showing a complete pathologic response in October 2018, in addition to an adjuvant radiation therapy. In May 2019, she presented an asymptomatic relapse on mediastinal lymph nodes detected on follow-up PET-CT scan and confirmed on biopsy with a negative PD-L1. The patient received 6 cycles of gemcitabine-carboplatin until October 2019.

She then started developing pruritic skin lesions over her scalp, face, neck, shoulders, and hands, that were exacerbated on sun exposure. She reported no systemic symptoms other than mild myalgia but no weakness.

**Figure 1:**
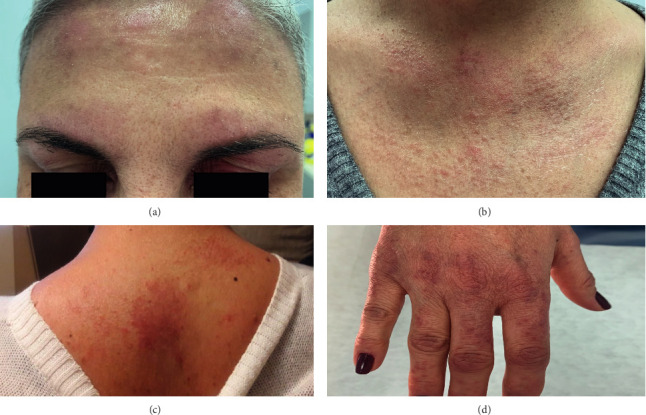
Clinical presentation with periorbital heliotrope rash (a), V-sign (b), shawl sign (c), Gottron's papules (d).

Skin examination revealed bilateral erythematous scaly patches on her forehead, periorbital violaceous erythema, and diffuse scales on her scalp. She also had erythematous coalescent papules on her upper chest and upper back and erythematous-violaceous papules over the dorsum of her hands and fingers (Figure 1). Differential diagnosis included DM, cutaneous lupus erythematosus, actinic lichen planus, and polymorphous light eruption. No muscular weakness was noted on physical examination.

A complete blood test revealed normal blood count and renal and liver function tests. She had no elevation in creatine kinase (CK) and lactate dehydrogenase (LDH) levels. Antinuclear antibodies (ANA), ANA blot, complement levels, and myositis-related antibodies were all negative. Chest X-ray showed no abnormalities.

**Figure 2:**
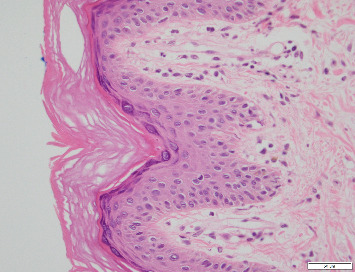
Histopathology ×40, focal vacuolar changes of the basal cell layer.

**Figure 3:**
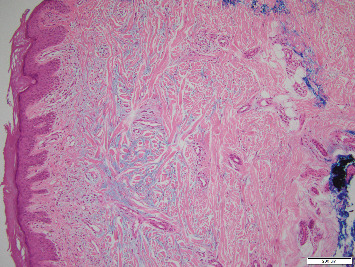
Increased interstitial mucin on histopathology alcian blue ×10.

Skin biopsies were done from 2 lesions on her hand and on her upper chest. Histopathology revealed confluent compact hyperkeratosis, mild hypergranulosis, mild papillomatous epidermal hyperplasia, focal vacuolar changes of the basal cell layer, and a superficial perivascular lymphocytic infiltrate with increased interstitial mucin (Figures 2 and 3). These findings were consistent with an interface dermatitis. Clinicopathologic correlation led to the diagnosis of amyopathic DM.

A control PET-CT scan done in February 2020 revealed a complete remission.

**Figure 4:**
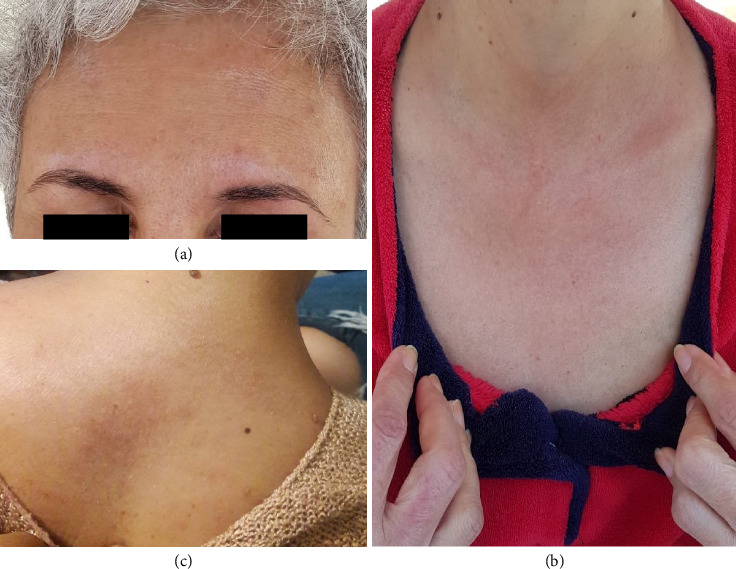
1 month after treatment with hydroxychloroquine and topical corticosteroids, clearance, or almost clearance of the lesions on the: face (a), décolleté (b), upper back (c).

The patient was started on hydroxychloroquine 200 mg per day and topical corticosteroids with a marked clinical improvement (Figure 4). In October 2020, the patient was stable with no muscular symptoms requiring muscle biopsy nor other systemic symptoms. Blood tests, including CK, and follow-up PET-CT scan remained negative.

## 3. Discussion

DM is a rare inflammatory myopathy characterized by classical cutaneous findings and proximal muscle weakness that could follow skin involvement by many years [2]. Typical skin lesions such as heliotrope rash, Gottron's papules, or Gottron's sign, are necessary to make the diagnosis [2]. Other characteristic findings include V-sign, Shawl sign, scaly scalp dermatitis, and periungual changes such as telangiectasias [2]. Skin biopsy is not required for the diagnosis when the patient presents with typical skin lesions and muscle involvement. When needed, histopathology is characterized by vacuolar alteration of the basal cell layer, mucin deposition, and perivascular lymphocytic infiltrate [3]. In addition, myositis-specific antibodies such as Mi-2, TIF1, MDA5, and Jo-1, are not always positive [2].

Our patient presented with characteristic cutaneous manifestations of DM, with absent muscular involvement, negative antibodies, and a histopathological diagnosis of interface vacuolar dermatitis. This presentation was consistent with the diagnosis of amyopathic DM, also known as dermatomyositis sine myositis, which represents 10%–30% of DM population with an incidence of 2 per 1 million persons [1]. This disease is characterized by the absence of subjective muscle weakness for at least 6 months following the diagnosis with normal results of myositis testing (CK, electromyography, MRI, or muscle biopsy) [4, 5].

Most cases are usually idiopathic, but DM can present as a paraneoplastic syndrome in 13%–33% of cases [4]. Malignancy generally occurs within 5 years of the diagnosis, or precedes it by 2 years, rarely more [5]. Older age is a risk factor for increased risk of neoplasia in the first 2 years [5]. In addition, negative ANA, positive antitranscription intermediary factor 1 gamma (TIF-1) and antinuclear matrix protein 2 (NXP2), and elevated erythrocyte sedimentation rate are associated with an increased risk of cancer [4]. However, studies have shown that no significant difference in cancer risk exists between amyopathic and myopathic DM [4, 5].

Breast cancer is the most common cancer worldwide and in Lebanon [6]. It is a rare diagnosis in patients with DM; however, it is more prevalent in patients with amyopathic DM than in those with myopathic form [1, 7]. In a review by Hendren et al. in 2017, most of the patients were diagnosed with breast cancer before the diagnosis of DM with an association with stage III or IV disease but without a predominance of a specific hormone receptor status [7].

In the case of amyopathic DM and breast cancer, we searched the PubMed database with the following terms: ([breast cancer] OR [breast neoplasm] OR [breast malignancy]) AND ([amyopathic dermatomyositis] OR [dermatomyositis sine myositis]). A total of 14 patients were included (Table 1). All patients were females with a mean age at diagnosis of 57 (range: 39–80 years). Breast cancer diagnoses ranged from 5 years prior to 1 year following amyopathic DM onset. In 3 patients, amyopathic DM was concomitant a relapse of their known breast cancer. In fact, in addition to age-appropriate cancer workup, in patients with underlying cancer, skin manifestations are a good marker of its activity, cutaneous lesions' recurrence usually indicates a malignancy relapse [7]. Therefore, patients must be evaluated and followed-up closely [7]. Our patient had amyopathic DM despite complete remission of her breast cancer, 2.5 years after initial diagnosis, confirmed on her normal PET-CT scans, with no evidence of other underlying malignancy.

In addition to photoprotection, topical corticosteroids and oral hydroxychloroquine are used to treat cutaneous manifestations of patients with amyopathic DM [1, 7]. Second-line therapy includes immunosuppressive agents such as methotrexate or mycophenolate mofetil [1]. High-dose oral corticosteroids (1 mg/kg) for 2–4 weeks are the mainstay of treatment when muscular involvement is present; they are less effective on skin lesions [1, 3, 7]. Furthermore, resolution of the paraneoplastic symptoms can sometimes be achieved when treating the underlying cancer [8, 9, 15]. Our patient was followed-up regularly and closely, with a very good response of her skin manifestations on hydroxychloroquine and topical corticosteroids.

## Figures and Tables

**Table 1:** Characteristics and treatment of patients with breast cancer and amyopathic dermatomyositis.

Author/year	Gender Age (years)	Time from ADM till Dx of BC	Treatment of breast cancer	Treatment ADM
Stonecipher et al. 1993 [8]	F 55	N/R (close temporal association)	Surgery	Improvement after surgery
Cosnes et al. 1995 [9]	N/R N/R	N/R	N/R	Resolution with the treatment of cancer
Dawkins et al. 1998 [10]	F 70	N/R	N/R	Short-term low dose prednisone, TCS
Goyal and Nousari 1999 [11]	F 39	Concomitant of BC relapse	Surgery	Hydroxychloroquine 200 mg bid, prednisone
El-Azhary and Pakzad 2002 [12]	F 76	−5 years	N/R	N/R
Blanes et al. 2005 [13]	F 59	Concomitant of BC relapse	Surgery, CT, RT, hormonotherapy	Resolution with the treatment of cancer
Aslanidis et al. 2006 [14]	F 39	−7 months	Surgery, adjuvant CT, tamoxifen; CT 2 years later for cancer relapse	Prednisolone 40 mg/d; then recurrence on cancer relapse and treatment with hydroxychloroquine and IVIg
Pusceddu et al. 2008 [15]	F 50	N/R	CT	Complete resolution with CT for BC
Park et al. 2008 [16]	F 45	Before the dx of BC (time N/R)	Surgery + adjuvant CT (cyclophosphamide, epirubicin, and fluorouracil)	Azathioprine + prednisone for concomitant ILD
Mebazaa et al. 2011 [17]	F 49	−1 year	Surgery, RT, CT	Corticosteroids
Yasar et al. 2012 [18]	F 57	+1 year	Surgery (lumpectomy), radiation	Hydroxychloroquine 200 mg bid + TCS
Ogawa et al. 2016 [19]	F 63	−2.5 years	CT (adriamycin and cyclophosphamide) trastuzumab, followed by radiation	Topical tacrolimus
Udkoff and Cohen 2016 [1]	F 80	+0.5 year	Surgery + adjuvant CT + radiation	Hydroxychloroquine 200 mg bid
Bowerman et al. 2020 [5]	N/R N/R	N/R	N/R	N/R
Inaguma et al. 2020 [20]	F 74	Concomitant of BC diagnosis	Neoadjuvant CT + total mastectomy	Prednisolone 20 mg

Abbreviations: ADM, amyopathic dermatomyositis; BC, breast cancer; CT, chemotherapy; Dx, diagnosis; F, female; ILD, interstitial lung disease; N/R, not reported; RT, radiotherapy; TCS, topical corticosteroids.
